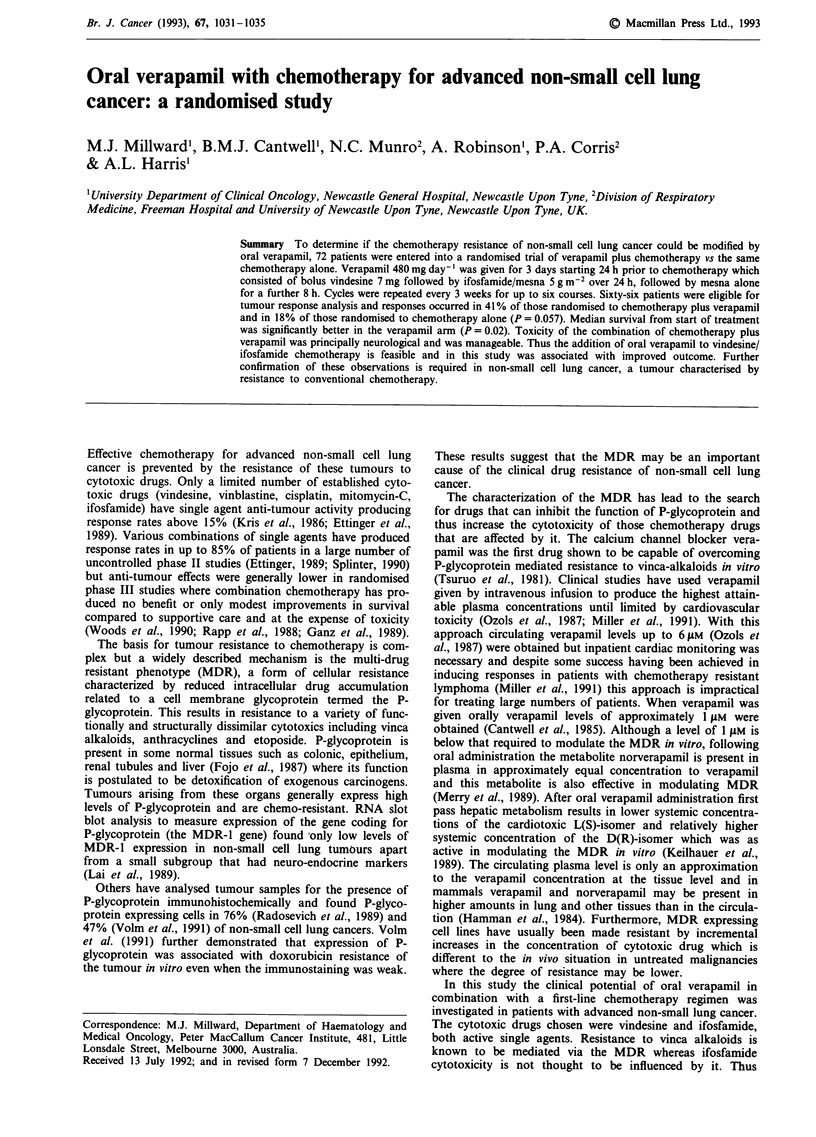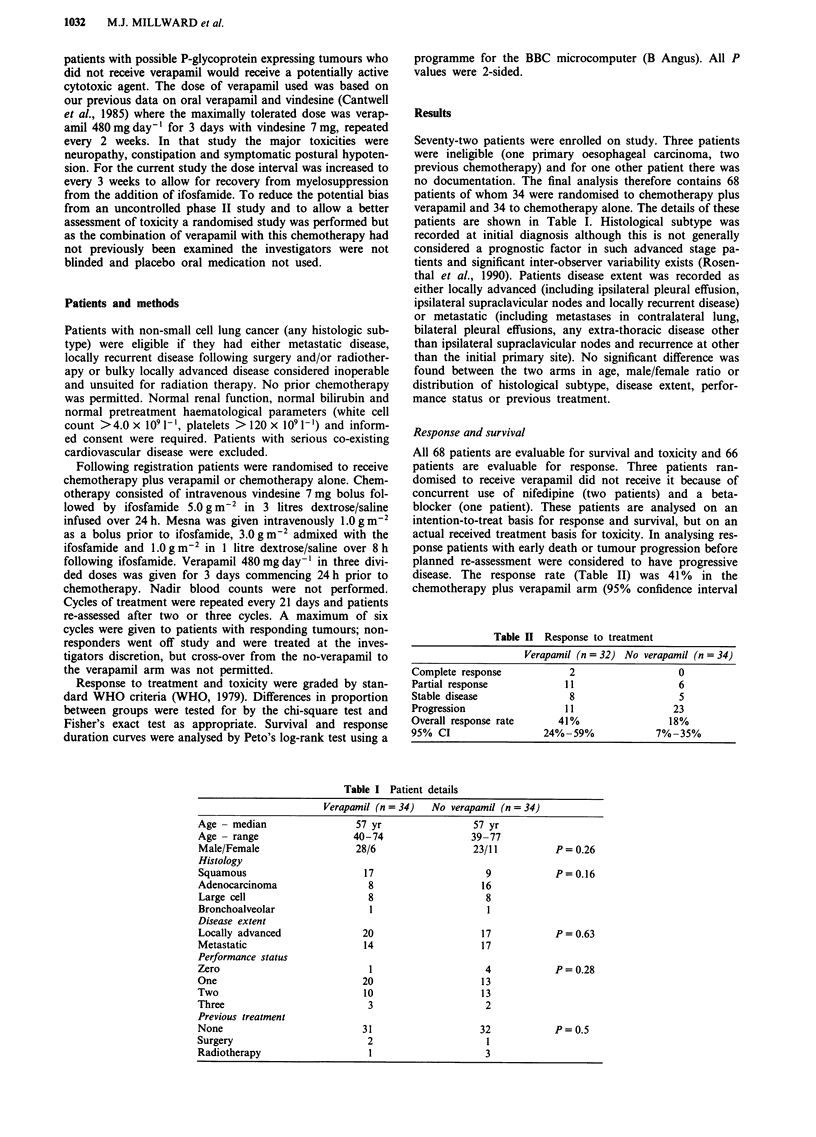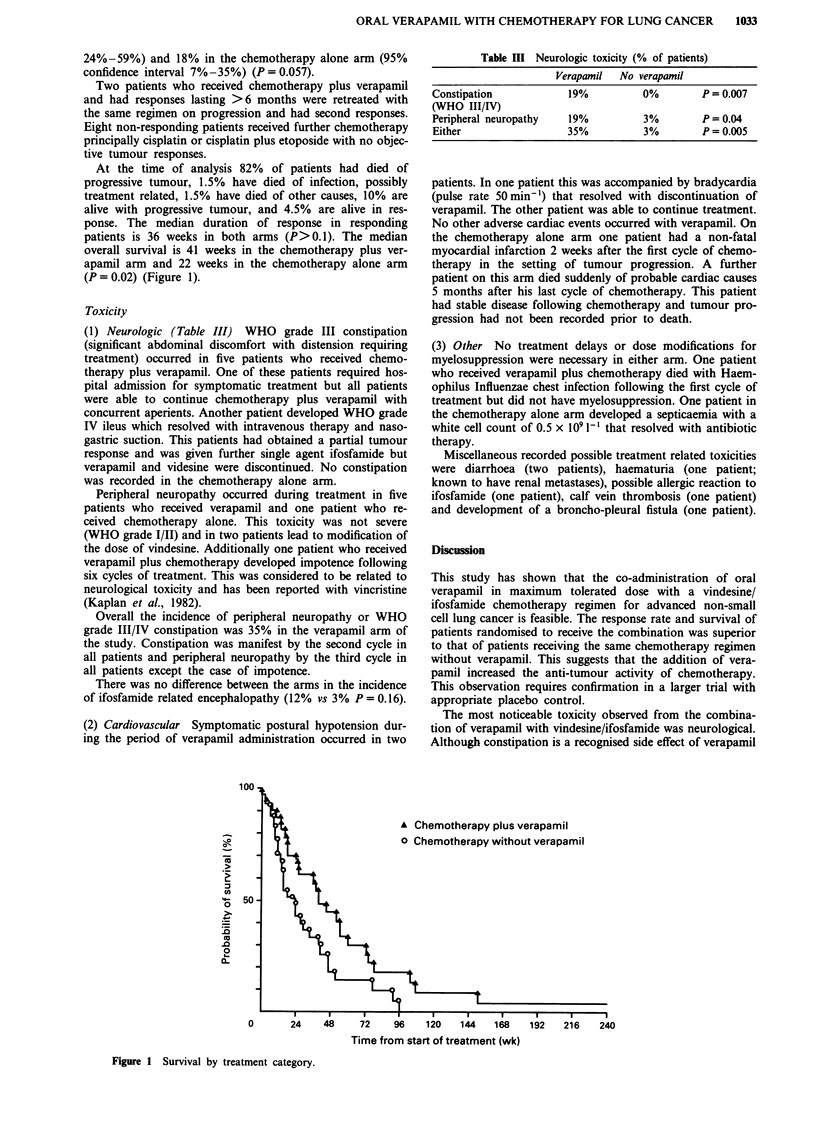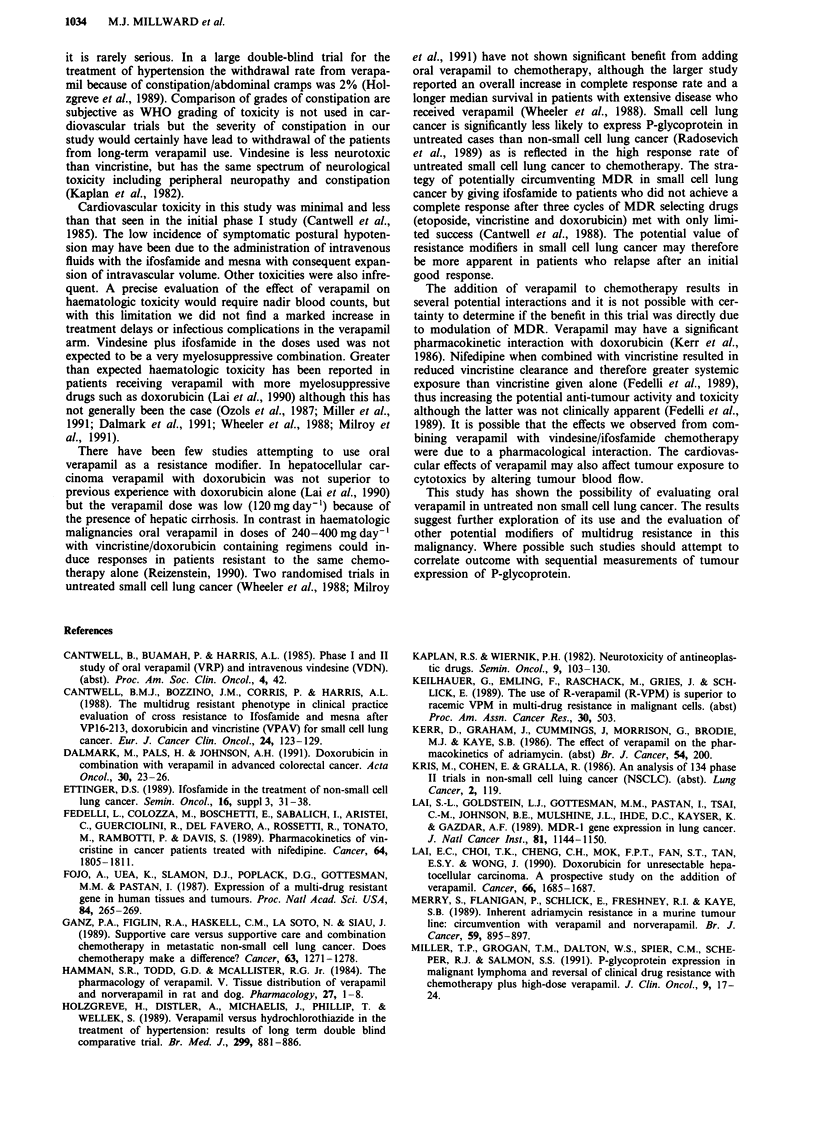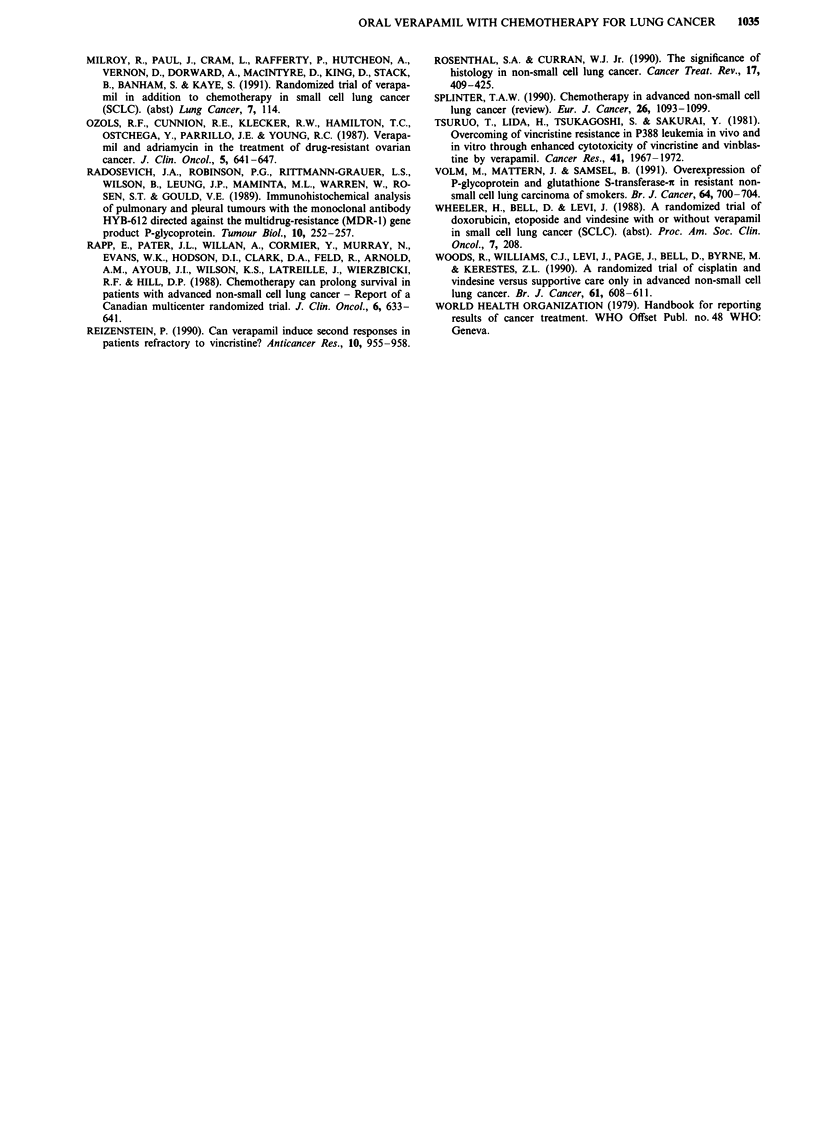# Oral verapamil with chemotherapy for advanced non-small cell lung cancer: a randomised study.

**DOI:** 10.1038/bjc.1993.189

**Published:** 1993-05

**Authors:** M. J. Millward, B. M. Cantwell, N. C. Munro, A. Robinson, P. A. Corris, A. L. Harris

**Affiliations:** University Department of Clinical Oncology, Newcastle General Hospital, Newcastle Upon Tyne, UK.

## Abstract

To determine if the chemotherapy resistance of non-small cell lung cancer could be modified by oral verapamil, 72 patients were entered into a randomised trial of verapamil plus chemotherapy vs the same chemotherapy alone. Verapamil 480 mg day-1 was given for 3 days starting 24 h prior to chemotherapy which consisted of bolus vindesine 7 mg followed by ifosfamide/mesna 5 g m-2 over 24 h, followed by mesna alone for a further 8 h. Cycles were repeated every 3 weeks for up to six courses. Sixty-six patients were eligible for tumour response analysis and responses occurred in 41% of those randomised to chemotherapy plus verapamil and in 18% of those randomised to chemotherapy alone (P = 0.057). Median survival from start of treatment was significantly better in the verapamil arm (P = 0.02). Toxicity of the combination of chemotherapy plus verapamil was principally neurological and was manageable. Thus the addition of oral verapamil to vindesine/ifosfamide chemotherapy is feasible and in this study was associated with improved outcome. Further confirmation of these observations is required in non-small cell lung cancer, a tumour characterised by resistance to conventional chemotherapy.


Br. J. Cancer (1993), 67, 1031-1035 © Macmillan Press Ltd., 1993

### Oral verapamil with chemotherapy for advanced non-small cell lung cancer: a randomised study

M.J. Millward[1], B.M.J. Cantwell[1], N.C. Munro[2], A. Robinson[1], P.A. Corris[2] & A.L. Harris[1]

[1]*University Department of Clinical Oncology, Newcastle General Hospital, Newcastle Upon Tyne,* [2]*Division of Respiratory Medicine, Freeman Hospital and University of Newcastle Upon Tyne, Newcastle Upon Tyne, UK.*

**Summary** To determine if the chemotherapy resistance of non-small cell lung cancer could be modified by oral verapamil, 72 patients were entered into a randomised trial of verapamil plus chemotherapy *vs* the same chemotherapy alone. Verapamil 480 mg day$^{-1}$ was given for 3 days starting 24 h prior to chemotherapy which consisted of bolus vindesine 7 mg followed by ifosfamide/mesna 5 g m$^{-2}$ over 24 h, followed by mesna alone for a further 8 h. Cycles were repeated every 3 weeks for up to six courses. Sixty-six patients were eligible for tumour response analysis and responses occurred in 41% of those randomised to chemotherapy plus verapamil and in 18% of those randomised to chemotherapy alone ($P = 0.057$). Median survival from start of treatment was significantly better in the verapamil arm ($P = 0.02$). Toxicity of the combination of chemotherapy plus verapamil was principally neurological and was manageable. Thus the addition of oral verapamil to vindesine/ifosfamide chemotherapy is feasible and in this study was associated with improved outcome. Further confirmation of these observations is required in non-small cell lung cancer, a tumour characterised by resistance to conventional chemotherapy.

Effective chemotherapy for advanced non-small cell lung cancer is prevented by the resistance of these tumours to cytotoxic drugs. Only a limited number of established cytotoxic drugs (vindesine, vinblastine, cisplatin, mitomycin-C, ifosfamide) have single agent anti-tumour activity producing response rates above 15% (Kris *et al.*, 1986; Ettinger *et al.*, 1989). Various combinations of single agents have produced response rates in up to 85% of patients in a large number of uncontrolled phase II studies (Ettinger, 1989; Splinter, 1990) but anti-tumour effects were generally lower in randomised phase III studies where combination chemotherapy has produced no benefit or only modest improvements in survival compared to supportive care and at the expense of toxicity (Woods *et al.*, 1990; Rapp *et al.*, 1988; Ganz *et al.*, 1989).

The basis for tumour resistance to chemotherapy is complex but a widely described mechanism is the multi-drug resistant phenotype (MDR), a form of cellular resistance characterized by reduced intracellular drug accumulation related to a cell membrane glycoprotein termed the P-glycoprotein. This results in resistance to a variety of functionally and structurally dissimilar cytotoxics including vinca alkaloids, anthracyclines and etoposide. P-glycoprotein is present in some normal tissues such as colonic, epithelium, renal tubules and liver (Fojo *et al.*, 1987) where its function is postulated to be detoxification of exogenous carcinogens. Tumours arising from these organs generally express high levels of P-glycoprotein and are chemo-resistant. RNA slot blot analysis to measure expression of the gene coding for P-glycoprotein (the MDR-1 gene) found only low levels of MDR-1 expression in non-small cell lung tumours apart from a small subgroup that had neuro-endocrine markers (Lai *et al.*, 1989).

Others have analysed tumour samples for the presence of P-glycoprotein immunohistochemically and found P-glycoprotein expressing cells in 76% (Radosevich *et al.*, 1989) and 47% (Volm *et al.*, 1991) of non-small cell lung cancers. Volm *et al.* (1991) further demonstrated that expression of P-glycoprotein was associated with doxorubicin resistance of the tumour *in vitro* even when the immunostaining was weak.

Correspondence: M.J. Millward, Department of Haematology and Medical Oncology, Peter MacCallum Cancer Institute, 481, Little Lonsdale Street, Melbourne 3000, Australia.
Received 13 July 1992; and in revised form 7 December 1992.

These results suggest that the MDR may be an important cause of the clinical drug resistance of non-small cell lung cancer.

The characterization of the MDR has lead to the search for drugs that can inhibit the function of P-glycoprotein and thus increase the cytotoxicity of those chemotherapy drugs that are affected by it. The calcium channel blocker verapamil was the first drug shown to be capable of overcoming P-glycoprotein mediated resistance to vinca-alkaloids *in vitro* (Tsuruo *et al.*, 1981). Clinical studies have used verapamil given by intravenous infusion to produce the highest attainable plasma concentrations until limited by cardiovascular toxicity (Ozols *et al.*, 1987; Miller *et al.*, 1991). With this approach circulating verapamil levels up to 6 μM (Ozols *et al.*, 1987) were obtained but inpatient cardiac monitoring was necessary and despite some success having been achieved in inducing responses in patients with chemotherapy resistant lymphoma (Miller *et al.*, 1991) this approach is impractical for treating large numbers of patients. When verapamil was given orally verapamil levels of approximately 1 μM were obtained (Cantwell *et al.*, 1985). Although a level of 1 μM is below that required to modulate the MDR *in vitro*, following oral administration the metabolite norverapamil is present in plasma in approximately equal concentration to verapamil and this metabolite is also effective in modulating MDR (Merry *et al.*, 1989). After oral verapamil administration first pass hepatic metabolism results in lower systemic concentrations of the cardiotoxic L(S)-isomer and relatively higher systemic concentration of the D(R)-isomer which was as active in modulating the MDR *in vitro* (Keilhauer *et al.*, 1989). The circulating plasma level is only an approximation to the verapamil concentration at the tissue level and in mammals verapamil and norverapamil may be present in higher amounts in lung and other tissues than in the circulation (Hamman *et al.*, 1984). Furthermore, MDR expressing cell lines have usually been made resistant by incremental increases in the concentration of cytotoxic drug which is different to the *in vivo* situation in untreated malignancies where the degree of resistance may be lower.

In this study the clinical potential of oral verapamil in combination with a first-line chemotherapy regimen was investigated in patients with advanced non-small lung cancer. The cytotoxic drugs chosen were vindesine and ifosfamide, both active single agents. Resistance to vinca alkaloids is known to be mediated via the MDR whereas ifosfamide cytotoxicity is not thought to be influenced by it. Thus patients with possible P-glycoprotein expressing tumours who did not receive verapamil would receive a potentially active cytotoxic agent. The dose of verapamil used was based on our previous data on oral verapamil and vindesine (Cantwell et al., 1985) where the maximally tolerated dose was verapamil 480 mg day$^{-1}$ for 3 days with vindesine 7 mg, repeated every 2 weeks. In that study the major toxicities were neuropathy, constipation and symptomatic postural hypotension. For the current study the dose interval was increased to every 3 weeks to allow for recovery from myelosuppression from the addition of ifosfamide. To reduce the potential bias from an uncontrolled phase II study and to allow a better assessment of toxicity a randomised study was performed but as the combination of verapamil with this chemotherapy had not previously been examined the investigators were not blinded and placebo oral medication not used.

### Patients and methods

Patients with non-small cell lung cancer (any histologic subtype) were eligible if they had either metastatic disease, locally recurrent disease following surgery and/or radiotherapy or bulky locally advanced disease considered inoperable and unsuited for radiation therapy. No prior chemotherapy was permitted. Normal renal function, normal bilirubin and normal pretreatment haematological parameters (white cell count $>4.0 \times 10^9 l^{-1}$, platelets $>120 \times 10^9 l^{-1}$) and informed consent were required. Patients with serious co-existing cardiovascular disease were excluded.

Following registration patients were randomised to receive chemotherapy plus verapamil or chemotherapy alone. Chemotherapy consisted of intravenous vindesine 7 mg bolus followed by ifosfamide 5.0 g m$^{-2}$ in 3 litres dextrose/saline infused over 24 h. Mesna was given intravenously 1.0 g m$^{-2}$ as a bolus prior to ifosfamide, 3.0 g m$^{-2}$ admixed with the ifosfamide and 1.0 g m$^{-2}$ in 1 litre dextrose/saline over 8 h following ifosfamide. Verapamil 480 mg day$^{-1}$ in three divided doses was given for 3 days commencing 24 h prior to chemotherapy. Nadir blood counts were not performed. Cycles of treatment were repeated every 21 days and patients re-assessed after two or three cycles. A maximum of six cycles were given to patients with responding tumours; non-responders went off study and were treated at the investigators discretion, but cross-over from the no-verapamil to the verapamil arm was not permitted.

Response to treatment and toxicity were graded by standard WHO criteria (WHO, 1979). Differences in proportion between groups were tested for by the chi-square test and Fisher's exact test as appropriate. Survival and response duration curves were analysed by Peto's log-rank test using a programme for the BBC microcomputer (B Angus). All $P$ values were 2-sided.

### Results

Seventy-two patients were enrolled on study. Three patients were ineligible (one primary oesophageal carcinoma, two previous chemotherapy) and for one other patient there was no documentation. The final analysis therefore contains 68 patients of whom 34 were randomised to chemotherapy plus verapamil and 34 to chemotherapy alone. The details of these patients are shown in Table I. Histological subtype was recorded at initial diagnosis although this is not generally considered a prognostic factor in such advanced stage patients and significant inter-observer variability exists (Rosenthal et al., 1990). Patients disease extent was recorded as either locally advanced (including ipsilateral pleural effusion, ipsilateral supraclavicular nodes and locally recurrent disease) or metastatic (including metastases in contralateral lung, bilateral pleural effusions, any extra-thoracic disease other than ipsilateral supraclavicular nodes and recurrence at other than the initial primary site). No significant difference was found between the two arms in age, male/female ratio or distribution of histological subtype, disease extent, performance status or previous treatment.

### *Response and survival*

All 68 patients are evaluable for survival and toxicity and 66 patients are evaluable for response. Three patients randomised to receive verapamil did not receive it because of concurrent use of nifedipine (two patients) and a beta-blocker (one patient). These patients are analysed on an intention-to-treat basis for response and survival, but on an actual received treatment basis for toxicity. In analysing response patients with early death or tumour progression before planned re-assessment were considered to have progressive disease. The response rate (Table II) was 41% in the chemotherapy plus verapamil arm (95% confidence interval

**Table II** Response to treatment

| | *Verapamil (n = 32)* | *No verapamil (n = 34)* |
|---|---|---|
| Complete response | 2 | 0 |
| Partial response | 11 | 6 |
| Stable disease | 8 | 5 |
| Progression | 11 | 23 |
| Overall response rate | 41% | 18% |
| 95% CI | 24%-59% | 7%-35% |

**Table I** Patient details

| | *Verapamil (n = 34)* | *No verapamil (n = 34)* | |
|---|---|---|---|
| Age - median | 57 yr | 57 yr | |
| Age - range | 40-74 | 39-77 | |
| Male/Female | 28/6 | 23/11 | $P = 0.26$ |
| *Histology* | | | |
| Squamous | 17 | 9 | $P = 0.16$ |
| Adenocarcinoma | 8 | 16 | |
| Large cell | 8 | 8 | |
| Bronchoalveolar | 1 | 1 | |
| *Disease extent* | | | |
| Locally advanced | 20 | 17 | $P = 0.63$ |
| Metastatic | 14 | 17 | |
| *Performance status* | | | |
| Zero | 1 | 4 | $P = 0.28$ |
| One | 20 | 13 | |
| Two | 10 | 13 | |
| Three | 3 | 2 | |
| *Previous treatment* | | | |
| None | 31 | 32 | $P = 0.5$ |
| Surgery | 2 | 1 | |
| Radiotherapy | 1 | 3 | |

24%–59%) and 18% in the chemotherapy alone arm (95% confidence interval 7%–35%) ($P = 0.057$).

Two patients who received chemotherapy plus verapamil and had responses lasting >6 months were retreated with the same regimen on progression and had second responses. Eight non-responding patients received further chemotherapy principally cisplatin or cisplatin plus etoposide with no objective tumour responses.

At the time of analysis 82% of patients had died of progressive tumour, 1.5% have died of infection, possibly treatment related, 1.5% have died of other causes, 10% are alive with progressive tumour, and 4.5% are alive in response. The median duration of response in responding patients is 36 weeks in both arms ($P > 0.1$). The median overall survival is 41 weeks in the chemotherapy plus verapamil arm and 22 weeks in the chemotherapy alone arm ($P = 0.02$) (Figure 1).

*Toxicity*

(1) *Neurologic (Table III)* WHO grade III constipation (significant abdominal discomfort with distension requiring treatment) occurred in five patients who received chemotherapy plus verapamil. One of these patients required hospital admission for symptomatic treatment but all patients were able to continue chemotherapy plus verapamil with concurrent aperients. Another patient developed WHO grade IV ileus which resolved with intravenous therapy and nasogastric suction. This patients had obtained a partial tumour response and was given further single agent ifosfamide but verapamil and videsine were discontinued. No constipation was recorded in the chemotherapy alone arm.

Peripheral neuropathy occurred during treatment in five patients who received verapamil and one patient who received chemotherapy alone. This toxicity was not severe (WHO grade I/II) and in two patients lead to modification of the dose of vindesine. Additionally one patient who received verapamil plus chemotherapy developed impotence following six cycles of treatment. This was considered to be related to neurological toxicity and has been reported with vincristine (Kaplan *et al.*, 1982).

Overall the incidence of peripheral neuropathy or WHO grade III/IV constipation was 35% in the verapamil arm of the study. Constipation was manifest by the second cycle in all patients and peripheral neuropathy by the third cycle in all patients except the case of impotence.

There was no difference between the arms in the incidence of ifosfamide related encephalopathy (12% *vs* 3% $P = 0.16$).

(2) *Cardiovascular* Symptomatic postural hypotension during the period of verapamil administration occurred in two patients. In one patient this was accompanied by bradycardia (pulse rate 50 $min^{-1}$) that resolved with discontinuation of verapamil. The other patient was able to continue treatment. No other adverse cardiac events occurred with verapamil. On the chemotherapy alone arm one patient had a non-fatal myocardial infarction 2 weeks after the first cycle of chemotherapy in the setting of tumour progression. A further patient on this arm died suddenly of probable cardiac causes 5 months after his last cycle of chemotherapy. This patient had stable disease following chemotherapy and tumour progression had not been recorded prior to death.

(3) *Other* No treatment delays or dose modifications for myelosuppression were necessary in either arm. One patient who received verapamil plus chemotherapy died with Haemophilus Influenzae chest infection following the first cycle of treatment but did not have myelosuppression. One patient in the chemotherapy alone arm developed a septicaemia with a white cell count of $0.5 \times 10^9 l^{-1}$ that resolved with antibiotic therapy.

Miscellaneous recorded possible treatment related toxicities were diarrhoea (two patients), haematuria (one patient; known to have renal metastases), possible allergic reaction to ifosfamide (one patient), calf vein thrombosis (one patient) and development of a broncho-pleural fistula (one patient).

**Table III** Neurologic toxicity (% of patients)

| | *Verapamil* | *No verapamil* | |
|---|---|---|---|
| Constipation (WHO III/IV) | 19% | 0% | $P = 0.007$ |
| Peripheral neuropathy | 19% | 3% | $P = 0.04$ |
| Either | 35% | 3% | $P = 0.005$ |

### Discussion

This study has shown that the co-administration of oral verapamil in maximum tolerated dose with a vindesine/ifosfamide chemotherapy regimen for advanced non-small cell lung cancer is feasible. The response rate and survival of patients randomised to receive the combination was superior to that of patients receiving the same chemotherapy regimen without verapamil. This suggests that the addition of verapamil increased the anti-tumour activity of chemotherapy. This observation requires confirmation in a larger trial with appropriate placebo control.

The most noticeable toxicity observed from the combination of verapamil with vindesine/ifosfamide was neurological. Although constipation is a recognised side effect of verapamil

**Figure 1** Survival by treatment category.

it is rarely serious. In a large double-blind trial for the treatment of hypertension the withdrawal rate from verapamil because of constipation/abdominal cramps was 2% (Holzgreve et al., 1989). Comparison of grades of constipation are subjective as WHO grading of toxicity is not used in cardiovascular trials but the severity of constipation in our study would certainly have lead to withdrawal of the patients from long-term verapamil use. Vindesine is less neurotoxic than vincristine, but has the same spectrum of neurological toxicity including peripheral neuropathy and constipation (Kaplan et al., 1982).

Cardiovascular toxicity in this study was minimal and less than that seen in the initial phase I study (Cantwell et al., 1985). The low incidence of symptomatic postural hypotension may have been due to the administration of intravenous fluids with the ifosfamide and mesna with consequent expansion of intravascular volume. Other toxicities were also infrequent. A precise evaluation of the effect of verapamil on haematologic toxicity would require nadir blood counts, but with this limitation we did not find a marked increase in treatment delays or infectious complications in the verapamil arm. Vindesine plus ifosfamide in the doses used was not expected to be a very myelosuppressive combination. Greater than expected haematologic toxicity has been reported in patients receiving verapamil with more myelosuppressive drugs such as doxorubicin (Lai et al., 1990) although this has not generally been the case (Ozols et al., 1987; Miller et al., 1991; Dalmark et al., 1991; Wheeler et al., 1988; Milroy et al., 1991).

There have been few studies attempting to use oral verapamil as a resistance modifier. In hepatocellular carcinoma verapamil with doxorubicin was not superior to previous experience with doxorubicin alone (Lai et al., 1990) but the verapamil dose was low ($120\,mg\,day^{-1}$) because of the presence of hepatic cirrhosis. In contrast in haematologic malignancies oral verapamil in doses of $240-400\,mg\,day^{-1}$ with vincristine/doxorubicin containing regimens could induce responses in patients resistant to the same chemotherapy alone (Reizenstein, 1990). Two randomised trials in untreated small cell lung cancer (Wheeler et al., 1988; Milroy et al., 1991) have not shown significant benefit from adding oral verapamil to chemotherapy, although the larger study reported an overall increase in complete response rate and a longer median survival in patients with extensive disease who received verapamil (Wheeler et al., 1988). Small cell lung cancer is significantly less likely to express P-glycoprotein in untreated cases than non-small cell lung cancer (Radosevich et al., 1989) as is reflected in the high response rate of untreated small cell lung cancer to chemotherapy. The strategy of potentially circumventing MDR in small cell lung cancer by giving ifosfamide to patients who did not achieve a complete response after three cycles of MDR selecting drugs (etoposide, vincristine and doxorubicin) met with only limited success (Cantwell et al., 1988). The potential value of resistance modifiers in small cell lung cancer may therefore be more apparent in patients who relapse after an initial good response.

The addition of verapamil to chemotherapy results in several potential interactions and it is not possible with certainty to determine if the benefit in this trial was directly due to modulation of MDR. Verapamil may have a significant pharmacokinetic interaction with doxorubicin (Kerr et al., 1986). Nifedipine when combined with vincristine resulted in reduced vincristine clearance and therefore greater systemic exposure than vincristine given alone (Fedelli et al., 1989), thus increasing the potential anti-tumour activity and toxicity although the latter was not clinically apparent (Fedelli et al., 1989). It is possible that the effects we observed from combining verapamil with vindesine/ifosfamide chemotherapy were due to a pharmacological interaction. The cardiovascular effects of verapamil may also affect tumour exposure to cytotoxics by altering tumour blood flow.

This study has shown the possibility of evaluating oral verapamil in untreated non small cell lung cancer. The results suggest further exploration of its use and the evaluation of other potential modifiers of multidrug resistance in this malignancy. Where possible such studies should attempt to correlate outcome with sequential measurements of tumour expression of P-glycoprotein.

### References

CANTWELL, B., BUAMAH, P. & HARRIS, A.L. (1985). Phase I and II study of oral verapamil (VRP) and intravenous vindesine (VDN). (abst). *Proc. Am. Soc. Clin. Oncol.*, **4**, 42.

CANTWELL, B.M.J., BOZZINO, J.M., CORRIS, P. & HARRIS, A.L. (1988). The multidrug resistant phenotype in clinical practice evaluation of cross resistance to Ifosfamide and mesna after VP16-213, doxorubicin and vincristine (VPAV) for small cell lung cancer. *Eur. J. Cancer Clin. Oncol.*, **24**, 123-129.

DALMARK, M., PALS, H. & JOHNSON, A.H. (1991). Doxorubicin in combination with verapamil in advanced colorectal cancer. *Acta Oncol.*, **30**, 23-26.

ETTINGER, D.S. (1989). Ifosfamide in the treatment of non-small cell lung cancer. *Semin. Oncol.*, **16**, suppl 3, 31-38.

FEDELLI, L., COLOZZA, M., BOSCHETTI, E., SABALICH, I., ARISTEI, C., GUERCIOLINI, R., DEL FAVERO, A., ROSSETTI, R., TONATO, M., RAMBOTTI, P. & DAVIS, S. (1989). Pharmacokinetics of vincristine in cancer patients treated with nifedipine. *Cancer*, **64**, 1805-1811.

FOJO, A., UEA, K., SLAMON, D.J., POPLACK, D.G., GOTTESMAN, M.M. & PASTAN, I. (1987). Expression of a multi-drug resistant gene in human tissues and tumours. *Proc. Natl Acad. Sci. USA*, **84**, 265-269.

GANZ, P.A., FIGLIN, R.A., HASKELL, C.M., LA SOTO, N. & SIAU, J. (1989). Supportive care versus supportive care and combination chemotherapy in metastatic non-small cell lung cancer. Does chemotherapy make a difference? *Cancer*, **63**, 1271-1278.

HAMMAN, S.R., TODD, G.D. & MCALLISTER, R.G. Jr. (1984). The pharmacology of verapamil. V. Tissue distribution of verapamil and norverapamil in rat and dog. *Pharmacology*, **27**, 1-8.

HOLZGREVE, H., DISTLER, A., MICHAELIS, J., PHILLIP, T. & WELLEK, S. (1989). Verapamil versus hydrochlorothiazide in the treatment of hypertension: results of long term double blind comparative trial. *Br. Med. J.*, **299**, 881-886.

KAPLAN, R.S. & WIERNIK, P.H. (1982). Neurotoxicity of antineoplastic drugs. *Semin. Oncol.*, **9**, 103-130.

KEILHAUER, G., EMLING, F., RASCHACK, M., GRIES, J. & SCHLICK, E. (1989). The use of R-verapamil (R-VPM) is superior to racemic VPM in multi-drug resistance in malignant cells. (abst) *Proc. Am. Assn. Cancer Res.*, **30**, 503.

KERR, D., GRAHAM, J., CUMMINGS, J, MORRISON, G., BRODIE, M.J. & KAYE, S.B. (1986). The effect of verapamil on the pharmacokinetics of adriamycin. (abst) *Br. J. Cancer*, **54**, 200.

KRIS, M., COHEN, E. & GRALLA, R. (1986). An analysis of 134 phase II trials in non-small cell luing cancer (NSCLC). (abst). *Lung Cancer*, **2**, 119.

LAI, S.-L., GOLDSTEIN, L.J., GOTTESMAN, M.M., PASTAN, I., TSAI, C.-M., JOHNSON, B.E., MULSHINE, J.L., IHDE, D.C., KAYSER, K. & GAZDAR, A.F. (1989). MDR-1 gene expression in lung cancer. *J. Natl Cancer Inst.*, **81**, 1144-1150.

LAI, E.C., CHOI, T.K., CHENG, C.H., MOK, F.P.T., FAN, S.T., TAN, E.S.Y. & WONG, J. (1990). Doxorubicin for unresectable hepatocellular carcinoma. A prospective study on the addition of verapamil. *Cancer*, **66**, 1685-1687.

MERRY, S., FLANIGAN, P., SCHLICK, E., FRESHNEY, R.I. & KAYE, S.B. (1989). Inherent adriamycin resistance in a murine tumour line: circumvention with verapamil and norverapamil. *Br. J. Cancer*, **59**, 895-897.

MILLER, T.P., GROGAN, T.M., DALTON, W.S., SPIER, C.M., SCHEPER, R.J. & SALMON, S.S. (1991). P-glycoprotein expression in malignant lymphoma and reversal of clinical drug resistance with chemotherapy plus high-dose verapamil. *J. Clin. Oncol.*, **9**, 17-24.

MILROY, R., PAUL, J., CRAM, L., RAFFERTY, P., HUTCHEON, A., VERNON, D., DORWARD, A., MACINTYRE, D., KING, D., STACK, B., BANHAM, S. & KAYE, S. (1991). Randomized trial of verapamil in addition to chemotherapy in small cell lung cancer (SCLC). (abst) *Lung Cancer*, **7**, 114.

OZOLS, R.F., CUNNION, R.E., KLECKER, R.W., HAMILTON, T.C., OSTCHEGA, Y., PARRILLO, J.E. & YOUNG, R.C. (1987). Verapamil and adriamycin in the treatment of drug-resistant ovarian cancer. *J. Clin. Oncol.*, **5**, 641-647.

RADOSEVICH, J.A., ROBINSON, P.G., RITTMANN-GRAUER, L.S., WILSON, B., LEUNG, J.P., MAMINTA, M.L., WARREN, W., ROSEN, S.T. & GOULD, V.E. (1989). Immunohistochemical analysis of pulmonary and pleural tumours with the monoclonal antibody HYB-612 directed against the multidrug-resistance (MDR-1) gene product P-glycoprotein. *Tumour Biol.*, **10**, 252-257.

RAPP, E., PATER, J.L., WILLAN, A., CORMIER, Y., MURRAY, N., EVANS, W.K., HODSON, D.I., CLARK, D.A., FELD, R., ARNOLD, A.M., AYOUB, J.I., WILSON, K.S., LATREILLE, J., WIERZBICKI, R.F. & HILL, D.P. (1988). Chemotherapy can prolong survival in patients with advanced non-small cell lung cancer - Report of a Canadian multicenter randomized trial. *J. Clin. Oncol.*, **6**, 633-641.

REIZENSTEIN, P. (1990). Can verapamil induce second responses in patients refractory to vincristine? *Anticancer Res.*, **10**, 955-958.

ROSENTHAL, S.A. & CURRAN, W.J. Jr. (1990). The significance of histology in non-small cell lung cancer. *Cancer Treat. Rev.*, **17**, 409-425.

SPLINTER, T.A.W. (1990). Chemotherapy in advanced non-small cell lung cancer (review). *Eur. J. Cancer*, **26**, 1093-1099.

TSURUO, T., LIDA, H., TSUKAGOSHI, S. & SAKURAI, Y. (1981). Overcoming of vincristine resistance in P388 leukemia in vivo and in vitro through enhanced cytotoxicity of vincristine and vinblastine by verapamil. *Cancer Res.*, **41**, 1967-1972.

VOLM, M., MATTERN, J. & SAMSEL, B. (1991). Overexpression of P-glycoprotein and glutathione S-transferase-π in resistant non-small cell lung carcinoma of smokers. *Br. J. Cancer*, **64**, 700-704.

WHEELER, H., BELL, D. & LEVI, J. (1988). A randomized trial of doxorubicin, etoposide and vindesine with or without verapamil in small cell lung cancer (SCLC). (abst). *Proc. Am. Soc. Clin. Oncol.*, **7**, 208.

WOODS, R., WILLIAMS, C.J., LEVI, J., PAGE, J., BELL, D., BYRNE, M. & KERESTES, Z.L. (1990). A randomized trial of cisplatin and vindesine versus supportive care only in advanced non-small cell lung cancer. *Br. J. Cancer*, **61**, 608-611.

WORLD HEALTH ORGANIZATION (1979). Handbook for reporting results of cancer treatment. WHO Offset Publ. no. 48 WHO: Geneva.